# Effects of Nicotinamide Adenine Dinucleotide on Older Patients with Heart Failure

**DOI:** 10.31083/j.rcm2508297

**Published:** 2024-08-21

**Authors:** Zuowei Pei, Min Dong, Xuyang Meng, Wei Yao, Ying Guo, Fang Wang

**Affiliations:** ^1^Department of Cardiology, Beijing Hospital, National Center of Gerontology, Institute of Geriatric Medicine, Chinese Academy of Medical Sciences, 100730 Beijing, China; ^2^Department of Cardiology, Central Hospital of Dalian University of Technology, 116033 Dalian, Liaoning, China; ^3^Department of Internal Medicine, Affiliated Zhong Shan Hospital of Dalian University, 116001 Dalian, Liaoning, China

**Keywords:** heart failure, NAD^+^, clinical study, adjuvant therapy

## Abstract

**Background:**

Heart failure (HF) is the main cause of death in middle-aged and older people and is characterized by high morbidity, high mortality, a high rehospitalization rate, and many high-risk groups. Nicotinamide adenine dinucleotide (NAD^+^) is widely present in the mitochondria of cardiomyocytes and maintains the redox balance in the body, which can effectively treat HF. We sought to evaluate whether NAD^+^ therapy has some clinical efficacy in patients with HF.

**Methods:**

Based on using conventional drugs to treat HF, patients (n = 60) were randomized 1:1 to saline and 50 mg NAD^+^ with 50 mL of normal saline for 7 days. The baseline characteristics of patients before and after treatment and cardiac function (N-terminal pro B-type natriuretic peptide (NT-proBNP) level and left ventricular ejection fraction (LVEF) value) were analyzed. Serological analysis (sirtuin-1 (SIRT1), sirtuin-3 (SIRT3), sirtuin-6 (SIRT6), reactive oxygen species (ROS), and endothelin) was also performed.

**Results:**

Among the 60 patients with HF who were treated with NAD^+^ for 7 days, the improvement rate in NT-proBNP levels and LVEF values was better than in the saline group, although not statistically significant. These patients were more likely to benefit from NAD^+^ because of higher levels of anti-oxidative stress (SIRT1, SIRT3, SIRT6, and ROS) and anti-endothelial injury (endothelin) than those in the saline control group.

**Conclusions:**

According to the results of this study, it is believed that 7 days of NAD^+^ injections has a positive effect on improving cardiac function, oxidative stress, and endothelial injury in patients with HF compared with the saline control.

**Clinical Trial Registration:**

Chinese Clinical Trial Registry (http://www.chictr.org.cn/) ChiCTR2300074326; retrospectively registered on 3 August 2023.

## 1. Introduction 

Heart failure (HF) poses a serious threat to human health and is characterized by high morbidity, high mortality, a high rehospitalization rate, and many high-risk groups. The American Heart Association/American College of Cardiology guidelines define HF as “a complex clinical syndrome caused by any structural or functional heart disease that affects the ability of the ventricles to fill and shoot blood” [1]. Benjamin *et al*. [2] reported that patients hospitalized for HF are at an increased risk of HF rehospitalization and cardiovascular death. One in eight deaths is due to HF, and the mortality rate within 5 years of diagnosis is as high as 50%, exceeding that of some malignancies [2].

Despite the increased number of drugs used to treat HF, the fatality rate remains high. The 2021 European Society of Cardiology guidelines recommend a combination of angiotensin-converting enzyme inhibitor (ACEI)/angiotensin-receptor neprilysin inhibitor, β-blockers, and aldosterone receptor antagonists (MRAs) as the primary treatment for chronic HF [3]. Both the previous “golden triangle” (β-blocker, ACEI/angiotensin II receptor blocker [ARB], and MRA) and the current “five golden flowers” (β-blocker, ACEI/ARB, MRA, and sodium-dependent glucose transporter 2 inhibitors) have limitations in clinical treatment [4, 5, 6]. Current drug therapy still cannot meet the needs of HF management, making it necessary to develop new treatment ideas and explore new treatment strategies.

Nicotinamide adenine dinucleotide (NAD^+^) is an important cofactor and a key metabolic enzyme substrate involved in redox reactions in the mitochondria, which is greatly significant in maintaining the balance of NAD^+^ *in vivo* for normal human metabolism [7]. Several studies have shown that NAD^+^ can inhibit inflammation and oxidative stress injury in endothelial cells, reduce apoptosis in microvascular endothelial cells, promote microangiogenesis, and improve microvascular injury caused by coronary microcirculation and myocardial ischemia-reperfusion [8]. Studies have also shown that the NAD^+^ concentration in the blood of patients with HF is significantly lower than in healthy people, and with an increase in age, NAD^+^ also has a trend of gradually decreasing [7, 9]. Therefore, stabilizing intracellular NAD^+^ levels through exogenous NAD^+^ supplementation is expected to be a therapeutic strategy for improving cardiac bioenergetics and function.

HF is the leading cause of hospitalization among older populations worldwide, with a high mortality rate and impact on the quality of life of patients [10, 11]. With the development of medical technology, various drugs have emerged to treat HF. However, the effectiveness of these drugs remains unsatisfactory. HF remains an intractable disease, with an increased burden of hospitalization and a continuous loss of health care expenditure [12]. Therefore, it is necessary to identify alternative and complementary treatment options. Thus, we sought to evaluate whether NAD^+^ therapy has some clinical efficacy in patients with HF.

## 2. Methods

### 2.1 Patients

Hospitalized patients older than 60 years of age, patients who met the diagnostic criteria for HF and were diagnosed with HF (New York Heart Association (NYHA) grades II–IV) with reduced ejection fraction (left ventricular ejection fraction [LVEF] ≤40%) or N-terminal pro B-type natriuretic peptide (NT-proBNP) ≥600 pg/mL, those who voluntarily participated in this study, and those who provided signed and dated written informed consent in accordance with the quality management standards for drug clinical trials and local laws were included.

Patients were excluded if any one of the following criteria was met: patients with high atrioventricular block, constrictive pericarditis, obstructive cardiomyopathy, or acute myocardial infarction; those complicated with malignant arrhythmia, pulmonary embolism, and other diseases; women who were pregnant or planning to become pregnant; women who were breastfeeding; patients with various tumors and HF caused by taking anti-tumor drugs; those with an NAD^+^, lactose intolerance, or lactose allergy; patients with secondary or primary unconsciousness, cognitive impairment, or mental behavior abnormalities; those with a history of severe allergy or infusion reaction; those with liver and kidney dysfunction; patients currently participating in clinical investigations of other drugs; other reasons for ineligibility for this clinical trial as determined by the investigator. The Beijing Hospital Ethics Committee approved the trial.

### 2.2 Trial Design

**Fig. 1.**
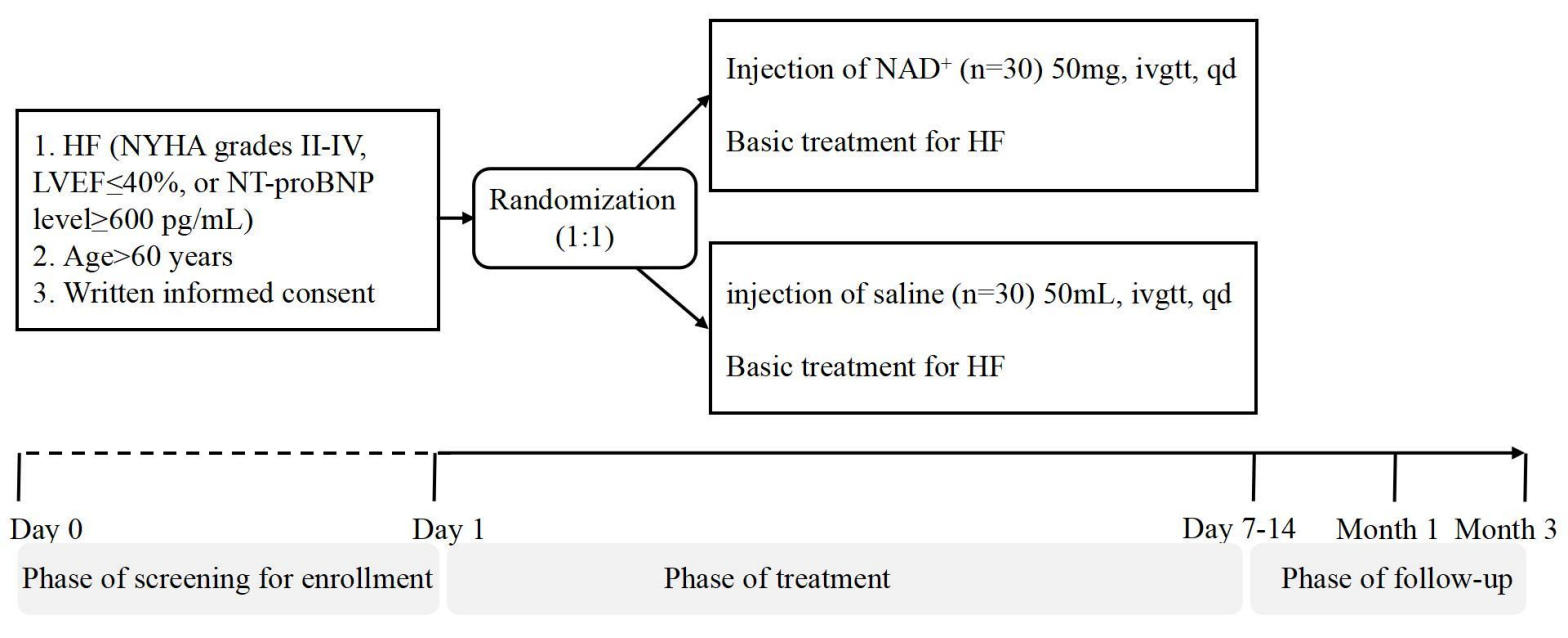
**Trial flow chart**. HF, heart failure; NYHA, New York Heart Association; LVEF, left ventricular ejection fraction; NT-proBNP, N-terminal pro B-type natriuretic peptide; NAD^+^, nicotinamide adenine dinucleotide.

The protective effect of NAD^+^ on patients with HF was studied in a single-center, prospective, randomized, controlled, double-blind clinical trial of hospitalized patients with HF (NYHA grades II–IV, LVEF ≤40%, or NT-proBNP level ≥600 pg/mL). Sixty hospitalized patients with HF were enrolled in this study and randomly divided into a saline control group or an NAD+ group at a 1:1 ratio (Fig. 1). In addition to the basic treatment for HF, patients in the saline control group received an intravenous injection of 50 mL of normal saline, whereas the NAD+ group received an intravenous injection of 50 mg NAD+ (Kaifeng Knature Pharmaceutical Co., Ltd., Henan, China; national drug approval number: H41024721) and 50 mL normal saline for 7 days. Three follow-up visits were conducted (2, 4, and 12 weeks after medication). Blood samples were collected at each follow-up to determine levels of NT-proBNP and other serum markers (including sirtuin-1 (SIRT1), sirtuin-3 (SIRT3), sirtuin-6 (SIRT6), reactive oxygen species (ROS), and endothelin (ET)). Experienced cardiologists performed the image analysis. LVEF was measured using the Simpson method with dual-plane endocardial surface imaging. The average value was calculated using three repeated measurements. The primary outcome measure was the improvement in NT-proBNP and LVEF values, and the secondary outcome measure was the composite endpoint of all-cause death and rehospitalization due to HF.

### 2.3 Definition of Post-treatment Response

The response after treatment was based on the definition of a composite endpoint, where echocardiography showed >5% improvement in LVEF and no major clinical events related to heart disease (including cardiovascular death or rehospitalization due to worsening HF). An absolute increase in LVEF ≥15%–20% or an absolute value ≥50% was defined as a post-treatment hyper-responder.

### 2.4 Biochemical Measurements

Fasting venous blood (3 mL) was collected from the two groups of patients at baseline and at 2, 4, and 12 weeks after treatments. Collected serum was prepared by centrifugation at 3600 rpm and 4 °C for 15 minutes, after which the supernatant was collected and used for SIRT1, SIRT3, SIRT6, ROS, and ET measurements using commercially available kits, according to the manufacturer’s instructions (Omnimabs, Alhambra, CA, USA).

### 2.5 Statistical Analysis

We described the baseline characteristics of the study population by treatment group using the mean and standard deviation, median, interquartile range, or percentage. Treatment group differences for changes in the LVEF, ET, SIRT1, SIRT6, and ROS were estimated using analysis of variance (ANOVA) of two-factor repeated measures fitted separately for each variable, and intra- and inter-group comparisons were performed. When the number of positive cases was <5, Fisher’s exact test was used to compare the rates and chi-square test results between the two groups of data. The comparison of NT-proBNP levels between the different groups and different follow-up visits was conducted using nonparametric two-factor ANOVA (Scheirer–Ray–Hare test). All statistical analyses were performed using SPSS software (version 25.0; IBM Corp., Armonk, NY, USA). A value of *p <* 0.05 was considered statistically significant.

## 3. Results

### 3.1 Baseline Characteristics

A total of 60 patients were screened, 2 of whom were excluded from the study without completing the medication; thus, 58 patients were included in the study (29 cases in the NAD^+^ group and 29 cases in the saline control group). Four patients died during follow-up, including three patients in the saline control group and one patient in the NAD^+^ group on days 65, 120, 60, and 42 after treatment, respectively. All of them were judged to have died during the natural course of the disease and that the medication did not cause their death.

Overall, baseline data were available for 58 patients. Table 1 shows significant group differences in baseline data were found only for hypertension. The number of hypertensive patients in the saline control group was higher than in the NAD^+^ group (*p* = 0.045). Since the prevalence of hypertension in HF patients was statistically different between the two groups, logistic regression analysis was performed with hypertension as the dependent variable and NT-proBNP improvement rate and LVEF improvement rate as the independent variables. The results showed that hypertension had no significant effect on NT-proBNP improvement rate ≥15% (*p* = 0.098), NT-proBNP improvement rate ≥30% (*p* = 0.087), and LVEF improvement rate ≥20% (*p* = 0.793). No statistically significant differences were observed in baseline characteristics affecting results such as age, sex, heart rate, comorbidities, and HF therapies between the saline control and NAD^+^ groups.

**Table 1.** **Baseline characteristics of the study population**.

		Saline control group (n = 29)	NAD^+^ group (n = 29)	*p*-value
Age, y		74.79 ± 16.24	73.03 ± 9.55	0.618
Male sex, n (%)		20 (69)	17 (58.6)	0.412
Systolic blood pressure, mmHg		128.1 ± 19.76	122.17 ± 13.64	0.189
Diastolic blood pressure, mmHg		76.03 ± 16.78	73.52 ± 10.98	0.502
BMI		24.43 ± 7.16	26.09 ± 4.95	0.310
Heart rate, beats/min (M (P_25_, P_75_))		80 (73, 88)	74 (65, 89)	0.196
HF characteristic				
	NYHA grade	3.10 ± 0.82	3.17 ± 0.76	0.740
	Hospitalized for HF in the past 12 months, n (%)	9 (31)	13 (44.8)	0.279
Comorbidity				
History, n (%)				
	Ischemic heart disease	14 (48.3)	12 (41.4)	0.597
	Non-ischemic heart disease	15 (51.7)	17 (58.6)	0.597
	Hypertension	12 (41.4)	5 (17.2)	0.043
	Diabetes mellitus	13 (43.3)	17 (40.0)	0.793
	Chronic kidney disease	20 (66.7)	20 (66.7)	1.000
	Coronary heart disease	10 (34.5)	6 (20.7)	0.240
	Old myocardial Infarction	17 (58.6)	18 (62.1)	0.788
	Three-branch lesion	20 (69)	20 (69)	1.000
	AF	12 (41.4)	9 (31)	0.412
	ICD	18 (62.1)	20 (69)	0.581
Heart failure therapies, n (%)				
	ACEI/ARB/ARNI	21 (72.4)	25 (86.2)	0.195
	Beta-blockers	22 (75.9)	26 (82.8)	0.164
	SGLT2-indicator	14 (48.3)	12 (41.4)	0.597
	Diuretics	21 (72.4)	26 (89.7)	0.094

Data are presented as mean ± standard deviation unless otherwise noted. AF, atrial fibrillation; ACEI, angiotensin-converting enzyme inhibitor; ARB, angiotensin II receptor blocker; ICD, implantable cardioverter-defibrillator; NYHA, New York Heart Association; HF, heart failure; NAD^+^, nicotinamide adenine dinucleotide; BMI, body mass index; M, median; P_25_, 25th percentile; P_75_, 75th percentile; ANRI, angiotensin receptor-neprilysin inhibitor; SGLT2, sodium-glucose cotransporter 2.

### 3.2 NAD^+^ Improved Cardiac Function

**Fig. 2.**
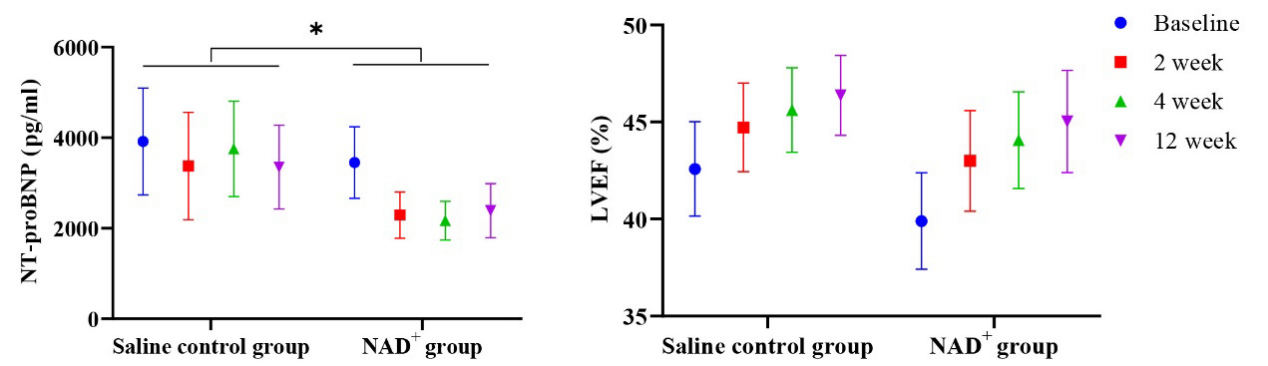
**Changes in NT-proBNP and LVEF values over time**. NT-proBNP levels in the saline control group and NAD^+^ group are decreased, whereas LVEF values in the saline control and NAD^+^ groups gradually improve. *p*-values delineate comparisons of NT-proBNP levels between the two groups over the three follow-up visits and are based on statistical methods using the Scheirer–Ray–Hare test, *p < 0.05. NAD^+^, nicotinamide adenine dinucleotide; LVEF, left ventricular ejection fraction; NT-proBNP, N-terminal pro B-type natriuretic peptide.

Table 2 and Fig. 2 compare cardiac function improvement between the two groups before and after treatment. The improvement in NT-proBNP and LVEF values at the three follow-up visits was higher in the NAD^+^ group than in the saline control group, although the difference was not statistically significant. After 12 weeks of follow-up, 11 patients (37.9%) in both the saline control and NAD^+^ groups had LVEF reactions, while five patients (17.2%) in the NAD^+^ group had LVEF hyper-reactions, which was higher than in the saline control group (10.3%).

**Table 2.** **Changes in NT-proBNP and LVEF levels and response before and after treatment**.

		Control group (n = 29)	NAD^+^ group (n = 29)		*p*-value
NT-proBNP improvement rate					
	Baseline NT-proBNP (pg/mL, M (P_25_, P_75_))	2242 (1135, 4200)	1536 (850, 4016)		0.308
	2WK-NT-proBNP improvement rate (%, M (P_25_, P_75_))	8.82 (–4.97, 84.82)	24.76 (–27.28, 47.40)		0.432
	4WK-NT-proBNP improvement rate (%, M (P_25_, P_75_))	12.45 (–30.82, 52.00)	29.75 (–16.59, 49.51)		0.514
	12WK-NT-proBNP improvement rate (%, M (P_25_, P_75_))	5.53 (–30.49, 61.80)	17.48 (–23.04, 58.69)		0.944
LVEF improvement rate					
	Baseline LVEF, % ($\bar{x}$ ± s)	42.59 ± 13.11	39.9 ± 13.35		0.442
	2WK-LVEF improvement rate, % ($\bar{x}$ ± s)	6.67 ± 14.50	8.78 ± 18.06		0.625
	4WK-LVEF improvement rate, % ($\bar{x}$ ± s)	9.87 ± 17.23	12.42 ± 20.50		0.610
	12WK-LVEF improvement rate, % ($\bar{x}$ ± s)	13.16 ± 23.47	14.44 ± 22.28		0.832
Post-treatment response					
	LVEF reaction rate, n (%)	11 (37.9)	11 (37.9)		1.000
	LVEF overreaction rate, n (%)	3 (10.3)	5 (17.2)		0.706
		Saline control group (n = 29)	NAD^+^ group (n = 29)	χ ^2^	*p*-value
NT-proBNP					
	Baseline-NT-proBNP (pg/mL, M (P_25_, P_75_))	2242 (1135.5, 4200)	1536 (850.3, 4016)	790.0	0.308
	2WK-NT-proBNP (pg/mL, M (P_25_, P_75_))	1591.0 (1115.0, 2981.0)	1295 (766.7, 2567.5)		
	4WK-NT-proBNP (pg/mL, M (P_25_, P_75_))	1909 (1028.5, 3806.0)	1260 (942.0, 3036.5)		
	12WK-NT-proBNP (pg/mL, M (P_25_, P_75_))	1761 (834.5, 3144.0)	1398 (824.5, 2359.5)		
χ ^2^		0.949		4.499	
*p*-value		0.418			0.035

Rate of NT-proBNP improvement = (baseline data – follow-up data)/baseline data. Rate of LVEF improvement = (follow-up data – baseline data)/baseline data. *p*-values delineate comparisons of the improvement rates of NT-proBNP and LVEF between two groups over three follow-up visits and are based on statistical methods using the independent two-sample *t*-test.* p*-values delineate post-treatment response based on statistical methods using Fisher’s exact test. *p*-values delineate comparisons of NT-proBNP levels between the two groups over follow-up visits and are based on statistical methods using the Scheirer–Ray–Hare test. NAD^+^, nicotinamide adenine dinucleotide; LVEF, left ventricular ejection fraction; NT-proBNP, N-terminal pro B-type natriuretic peptide; 2WK, 2-week follow-up; 4WK, 4-week follow-up; 12WK, 12-week follow-up; M, median; P_25_, 25th percentile; P_75_, 75th percentile.

No significant difference existed between the groups in NT-proBNP levels at baseline (*p* = 0.308). There was a statistically significant difference in the change trend of the NT-proBNP levels between the groups after medication (*p *= 0.035), and the improvement rate of NT-proBNP in the NAD^+^ group was more obvious than in the saline control group (Table 2). This finding further confirmed that NAD^+^ is key in reducing NT-proBNP levels.

### 3.3 NAD^+^ Inhibited Heart Oxidative Stress

**Fig. 3.**
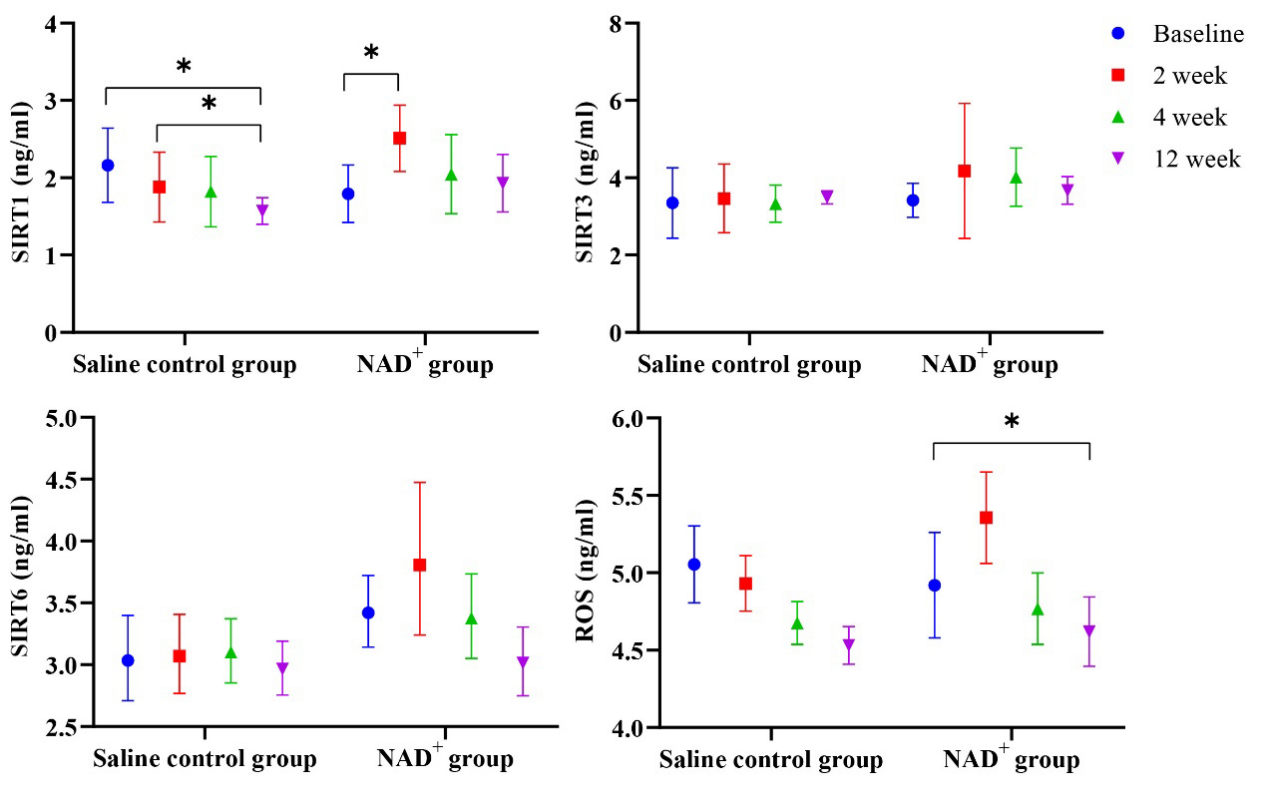
**Changes in SIRT1, SIRT6, and ROS over time**. The SIRT1, SIRT3, SIRT6, and ROS levels are higher in the NAD^+^ group than in the saline control group at the three follow-up visits. Over time, those aforementioned levels decreased in the NAD^+^ group, *p < 0.05. NAD^+^, nicotinamide adenine dinucleotide; SIRT1, sirtuin-1; SIRT3, sirtuin-3; SIRT6, sirtuin-6; ROS, reactive oxygen species.

Comparisons of serum SIRT1, SIRT3, SIRT6, and ROS levels between the two groups before and after treatment are shown in Table 3 and Fig. 3. After medication, SIRT1 levels in both groups changed, and the trend of change within each group was statistically different (*p *= 0.005), and the interaction was statistically different (*p* = 0.000). Owing to the statistical difference in time, a pairwise comparison was conducted. In the NAD^+^ group, SIRT1 levels were significantly higher than baseline at 2 weeks after treatment (*p* = 0.006), whereas, in the control group, there was a statistically significant difference between baseline and the 12-week follow-up (*p* = 0.000) and between the 4-week and 12-week follow-ups (*p* = 0.013).

**Table 3.** **Changes in the SIRT1, SIRT3, SIR6, and ROS values before and after treatment**.

		Saline control group (n = 29)	NAD^+^ group (n = 29)	F	*p*-value
SIRT1					
	Baseline SIRT1, ng/mL ($\bar{x}$ ± s)	2.28 ± 0.47	1.78 ± 0.39		
	2WK-SIRT1, ng/mL ($\bar{x}$ ± s)	1.98 ± 0.43	2.37 ± 0.36		
	4WK-SIRT1, ng/mL ($\bar{x}$ ± s)	1.91 ± 0.50	2.01 ± 0.43		
	12WK-SIRT1, ng/mL ($\bar{x}$ ± s)	1.57 ± 0.18	1.99 ± 0.35		
	Time			4.620	0.005
	Group			1.285	0.269
	Group*time			7.440	0.000
SIRT3					
	Baseline SIRT3, ng/mL ($\bar{x}$ ± s)	3.66 ± 0.74	3.35 ± 0.41		
	2WK-SIRT3, ng/mL ($\bar{x}$ ± s)	3.63 ± 0.66	3.52 ± 1.47		
	4WK-SIRT3, ng/mL ($\bar{x}$ ± s)	3.30 ± 0.56	3.89 ± 0.88		
	12WK-SIRT3, ng/mL ($\bar{x}$ ± s)	3.49 ± 0.18	3.64 ± 0.39		
	Time			0.076	0.923
	Group			0.212	0.650
	Group*time			1.950	0.156
SIRT6					
	Baseline SIRT6, ng/mL ($\bar{x}$ ± s)	3.05 ± 0.98	3.52 ± 0.62		
	2WK-SIRT6, ng/mL ($\bar{x}$ ± s)	3.15 ± 0.92	3.85 ± 1.24		
	4WK-SIRT6, ng/mL ($\bar{x}$ ± s)	3.18 ± 0.63	3.35 ± 0.81		
	12WK-SIRT6, ng/mL ($\bar{x}$ ± s)	3.00 ± 0.40	2.98 ± 0.46		
	Time			1.470	0.239
	Group			6.168	0.021
	Group*time			0.825	0.458
ROS					
	Baseline ROS, ng/mL ($\bar{x}$ ± s)	5.71 ± 1.24	5.35 ± 1.30		
	2WK-ROS, ng/mL ($\bar{x}$ ± s)	5.20 ± 1.06	5.69 ± 1.30		
	4WK-ROS, ng/mL ($\bar{x}$ ± s)	4.76 ± 0.69	4.99 ± 1.18		
	12WK-ROS, ng/mL ($\bar{x}$ ± s)	4.54 ± 0.52	4.75 ± 0.96		
	Time			4.260	0.008
	Group			0.380	0.544
	Group*time			0.741	0.531

*p*-values delineate comparisons of SIRT1, SIRT3, and ROS between the two groups over the three follow-up visits and are based on statistical methods using Fisher’s exact test. SIRT1, sirtuin-1; SIRT3, sirtuin-3; SIRT6, sirtuin-6; ROS, reactive oxygen species; NAD^+^, nicotinamide adenine dinucleotide; 2WK, 2-week follow-up; 4WK, 4-week follow-up; 12WK, 12-week follow-up.

In the NAD^+^ group, SIRT3 levels increased gradually, reaching a peak at the 4-week follow-up, and then decreased gradually at the 12-week follow-up but remained above the baseline level. SIRT3 levels in the saline control group did not increase.

SIRT6 values in the saline control and NAD^+^ groups were statistically different (*p* = 0.021); thus, NAD^+^ played a key role in increasing SIRT6 levels. In the NAD^+^ group, SIRT6 levels peaked at the 2-week follow-up, after which the levels gradually decreased. However, the elevated SIRT6 level in the saline control group was much lower than in the NAD^+^ group. The ROS values at the follow-up visits were statistically significant (*p *= 0.021).

### 3.4 NAD^+^ Improved Endothelial Function

A comparison of the serum ET levels between the two groups before and after treatment is shown in Table 4. The ET values at the follow-up visits were statistically significant between the two groups (*p <* 0.05), confirming the key role of NAD^+^ in anti-endothelial injury.

**Table 4.** **Change in ET values before and after treatment**.

	Saline control group (n = 29)	NAD^+^ group (n = 29)	*F*	*p*-value
ET				
Baseline ET, ng/mL ($\bar{x}$ ± s)	17.87 ± 3.76	18.17 ± 4.06		
2WK-ET, ng/mL ($\bar{x}$ ± s)	15.76 ± 3.09	14.92 ± 4.82		
4WK-ET, ng/mL ($\bar{x}$ ± s)	16.92 ± 2.14	15.06 ± 3.08		
12WK-ET, ng/mL ($\bar{x}$ ± s)	18.96 ± 0.98	17.43 ± 2.23		
Time			4.069	0.025
Group			5.965	0.023
Group*time			0.452	0.636

*p*-values delineate comparisons of ET values between the two groups over the three follow-up visits and are based on statistical methods using Fisher’s exact test. ET, endothelin; NAD^+^, nicotinamide adenine dinucleotide; 2WK, 2-week follow-up; 4WK, 4-week follow-up; 12WK, 12-week follow-up.

**Fig. 4.**
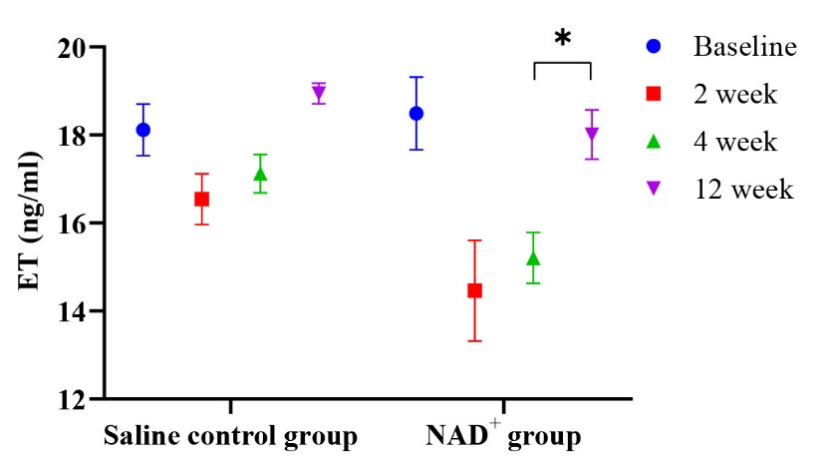
**Changes in the ET value over time**. At the 2-week follow-up, the ET level is lower in the NAD^+^ group than in the control group; with the decrease in the drug concentration, the ET level gradually increases, yet it is higher in the control group than in the NAD^+^ group, *p < 0.05. ET, endothelin; NAD^+^, nicotinamide adenine dinucleotide.

As shown in Fig. 4, ET levels in the NAD^+^ group were significantly lower than those in the saline control group during the second week of follow-up. The first two follow-up visits showed that NAD^+^ gradually reduced ET expression and then increased it at the last follow-up visit.

### 3.5 One-year Clinical Outcomes

We observed the clinical results for patients from March 2021 to September 2022 (Table 5), and there was no statistical difference in the observation indicators between the two groups. However, the 1-year survival rate was slightly higher in the NAD^+^ group than in the saline control group (96.6% versus 89.7%, *p* = 0.300).

**Table 5.** **One-year clinical outcomes of the entire cohort**.

	Control group (n = 29)	NAD^+^ group (n = 29)	*p*-value
Readmission and treatment, n (%)	9 (31.0)	10 (34.5)	0.780
Readmission due to worsening HF, n (%)	5 (17.2%)	5 (17.2%)	1.000
Readmission, time ($\bar{x}$ ± s)	0.24 ± 0.69	0.34 ± 0.67	0.564
Invasive manipulation, n (%)	13 (44.8)	8 (27.6)	0.172
Complete follow-up visit, n (%)	26 (89.7)	26 (89.7)	1.000
Survival, n (%)	26 (89.7)	28 (96.6)	0.300

Invasive procedures included percutaneous coronary intervention, artificial cardiac pacing, radiofrequency ablation, and invasive mechanical ventilation. NAD^+^, nicotinamide adenine dinucleotide; HF, heart failure.

## 4. Discussion

NAD^+^ is widely present in the mitochondria of myocardial tissue, and NAD^+^ activates the deacetylation activity of sirtuins, regulates the activity of numerous aging-related transcription factors, and intervenes in aging and aging-related diseases [9, 13, 14]. Several studies have shown that NAD^+^ has beneficial effects on cardiovascular diseases by regulating metabolism, maintaining redox homeostasis, and modulating immune responses [15, 16, 17]. Studies have found that NAD^+^ levels are significantly reduced in patients with HF [18, 19]. In a rat model of myocardial infarction, compared with the LCZ696-positive drug control group, using NAD^+^ improved some cardiac function and hemodynamic indexes and could further increase the left ventricular stroke output and systolic and diastolic blood pressure [20]. In the present clinical trial, we tested whether using exogenous NAD^+^ supplementation as a new adjuvant treatment for HF is possible.

NT-proBNP and LVEF are the most widely used laboratory indices for evaluating HF severity and prognosis. In our clinical trial, NT-proBNP levels improved in both groups, possibly because patients in both groups were rigorously treated with anti-HF drugs. However, the improvement was more obvious in the NAD^+^ group than in the saline control group, which better reflects that the therapeutic effect of NAD^+^ in patients with HF is independent of conventional medication for HF. Although the NT-proBNP level in the NAD^+^ group improved from baseline, no statistically significant difference was found over time. This finding may be due to the large dispersion of the data, short treatment course, and small sample size; similarly, due to short-term drug use, changes in the structure and function of the heart cannot be obvious over a short time. Moreover, the measurement of LVEF is subjective to a certain extent and is related to the experience of the tester. LVEF in the NAD^+^ group was improved, with no statistical difference between the groups. Studies have found that women may have lower NAD^+^ concentrations and benefit most from improved heart function [21, 22]. In our study, there was no statistical difference in gender between the two groups of patients, and whether women can benefit more from NAD^+^ treatment will be our major direction in the future.

Oxidative stress plays an important role in HF occurrence and progression [23]. The sirtuin family is also linked to several antioxidant and oxidative stress-related processes and functions [24]. Sirtuins are a family of seven enzymes (sirtuin1–7) involved in regulating many metabolic processes [25]. Sirtuin agonists are more convincing than existing deacetylase inhibitors for the treatment of cardiovascular diseases in terms of safety and efficacy and may have clinical value for treating multiple types of cardiovascular diseases [26]. Increasing the NAD^+^ level *in vivo* can activate sirtuins, which can significantly inhibit myocardial hyperacetylation and improve myocardial mitochondrial function [27]. Clemency *et al*. [28] found that sodium-glucose cotransporter 2 (SGL-2) inhibitor could inhibit oxidative stress by increasing the expression of SIRT1 and SIRT3 and decreasing the expression of SIRT6, thereby alleviating myocardial injury. In our clinical trial, the SIRT1, SIRT3, and SIRT6 levels increased at the 2-week follow-up after medication, but over time, the concentration of exogenous supplemental NAD^+^ gradually decreased *in vivo* and showed a trend of gradual decrease in the last two follow-up visits, which is also consistent with the metabolic process of intravenous drug use in the body. Furthermore, there was an interaction between SIRT1 in the saline control group and the NAD^+^ group, which confirmed that NAD^+^ plays a critical role in anti-oxidative stress. We observed that ROS levels increased in the NAD^+^ group at the 2-week follow-up visit, possibly due to the negative feedback reaction of inflammation caused by the strong antioxidant effect in the short term. However, those levels improved at later visits.

Increased levels of ET, the most potent vasoconstrictor, are produced by the pro-peptide precursor, large ET, through ET convertase [29]. ET in the peripheral blood of patients with HF can predict poor prognosis [30, 31, 32]. In this clinical trial, there were significant differences in ET levels between the saline control and NAD^+^ groups, which confirmed that NAD^+^ plays a more critical role in anti-endothelial injury.

Although there was no difference in the incidence of composite endpoint events (including all-cause death and readmission due to HF) during the 1-year follow-up period in this study, there was still a significant difference in mortality between the two groups. The survival rate of NAD^+^ was higher, and most patients who died were in the saline control group, usually 2–4 months after treatment. These results suggest that the use of NAD^+^ may delay the progression of HF and reduce short-term mortality. The reason why no statistical difference was found in the various clinical endpoints in this study may be related to the small number of study cases, the short duration of NAD^+^ administration, and the short follow-up time. Nevertheless, this study has expanded our ideas for exploring new treatments for patients with HF and confirmed their therapeutic effect on patients with HF based on molecular biology and echocardiography evaluation indicators. We also expect this study’s results to guide follow-up national multicenter, large-sample, prospective, randomized, double-blind controlled studies. We further confirmed the therapeutic effects of NAD^+^ in the HF population.

According to the product instructions, NAD^+^ occasionally has side effects such as dry mouth, nausea, dizziness, and palpitations. However, in this clinical trial, patients had no obvious complaints after 7 days of intravenously administering NAD^+^, but the long-term effect or safety of treatment still needs to be observed over a longer study period. In addition, the follow-up of participants after completing this clinical trial may be limited, making it difficult to assess long-term outcomes and treatment safety.

## 5. Limitations

Our study consisted of a small sample size from a single center, potentially limiting the generalizability of our findings to a broader population of HF patients. Future multicenter studies covering cohorts with different demographic and clinical characteristics would help validate our findings and enhance external validity. In addition, we did not perform cardiovascular magnetic resonance to assess patients with HF more comprehensively. We did not discuss the pharmacological background of HF patients further, and our future research direction will be to focus on their pharmacological background.

## 6. Conclusions

Among patients with HF, those injected with NAD^+^ for 7 days may benefit more from improved cardiac function, levels of anti-oxidative stress, and endothelial injury than those receiving saline. A prospective observational study will determine the efficacy and safety of NAD^+^, a promising adjuvant therapy for patients with HF.

## Data Availability

The data underlying this study are not publicly available as they include patient-level data, but are available from the corresponding author on reasonable request.
